# Immunologic Function and Molecular Insight of Recombinant Interleukin-18

**DOI:** 10.1371/journal.pone.0160321

**Published:** 2016-08-02

**Authors:** Jirakrit Saetang, Aekkachai Puseenam, Niran Roongsawang, Supayang Piyawan Voravuthikunchai, Surasak Sangkhathat, Varomyalin Tipmanee

**Affiliations:** 1 Department of Biomedical Sciences, Faculty of Medicine, Prince of Songkla University, Songkhla, 90110, Thailand; 2 Graduate School, Prince of Songkla University, Songkhla, 90110, Thailand; 3 Microbial Cell Factory Laboratory, Bioresources Technology Unit, National Center for Genetic Engineering and Biotechnology, National Science and Technology Development Agency, 113 Thailand Science Park, Phahonyothin Road, Khlong Nueng, Khlong Luang, Pathum Thani, 12120, Thailand; 4 Department of Microbiology and Natural Product Research Center of Excellence, Faculty of Science, Prince of Songkla University, Songkhla, 90110, Thailand; 5 Department of Surgery, Faculty of Medicine, Prince of Songkla University, Songkhla, 90110, Thailand; Oak Ridge National Laboratory, UNITED STATES

## Abstract

In recent years, cytokine-mediated therapy has emerged as further advance alternative in cancer therapy. Interleukin-18 (IL-18) has exhibited interesting anti-cancer properties especially when combined with IL-12. We engineered IL-18 in order to improve its activity using single point mutagenesis. IL-18 mutants were constructed according to binding residues and polarity which we tried to increase polarity in M33Q and M60Q, enhanced cationicity in E6K, and flexibility in T63A. All IL-18 proteins were expressed in *Pichia pastoris*, purified, and then measured the activity by treating with the NK-92MI cell line to evaluate interferon-γ (IFN-γ) stimulation. The E6K and T63A mutant forms showed higher activity with respect to native proteins at the concentration of 200 ng mL^-1^ by inducing the expression of IFN-γ, about factors of 9 and 4, respectively. Meanwhile, M33Q and M60Q had no significant activity to induce IFN-γ. Interestingly, the combination of E6K and T63A mutations could synergize the induction activity of IL-18 to be 16 times at 200 ng mL^-1^. Furthermore, molecular dynamics studies have elucidated the effect due to mutation on conformation of the binding site of IL-18. The results turn out that E6K provides structural perseverance against mutation, while M33Q and M60Q promote vivid overall change in protein conformation, especially at the binding site. For T63A, mutation yields small difference in structure but clearly increases structural flexibility. However, a small structural change was observed when T63A was combined with E6K. Our research resulted in a novel version of IL-18 which could be a new key candidate for cytokine-mediated therapy.

## Introduction

Cancer is a current public health concerns worldwide [1], with approximately 14 million new cases and 8.2 million cancer related deaths in 2012 [2]. This situation has led to increased research into new means of effective cancer control and therapy. One promising alternative of therapy is immunotherapy, a treatment type which exploits an immune system to facilitate cancer cell elimination and has been of major interest over past decades in both laboratory and clinical research [3]. The distinctive technique involves an application of monoclonal antibodies in cancer treatment through various mechanism, such as immune-mediated cell killing, specific effects on tumor microenvironment, or direct action on tumor cells which the last is the most interesting techniques in cancer therapy [4]. Moreover, the development of cancer vaccines, both therapeutic and preventive ones, have recently proven to be successful, so as to stimulate factors associated with improved survival rates in cancer patients [5,6], for example CD4^+^ T cells, CD8^+^ T cells and IFN- γ [6]. Apart from above-mentioned technique, non-specific immunotherapy concerns any indirect action to harm cancer cells. In this view, the action can be either to stimulate or switch an immunosuppressive context to anti-tumor progressive action in the tumor microenvironment through some cytokines [7].

Among the interesting cytokines regarding immunotherapy, interleukin-18 (IL-18), a cytokine species in the interleukin-1 family, has exhibited anti-cancer properties, via stimulation of natural killer cells (NK cells)[8] and cytotoxic T lymphocytes[9], as well as showing inhibitory effects on cancer cell growth and metastasis [10]. It is also able to encourage more efficient cancer cell killing through enhancing Fas-ligand expression in immune cells [11,12]. An animal study found fever was not present in IL-18 treated mice or rabbits although IL-18 is a known pro-inflammatory cytokine [13]. In addition, chills and fevers were rare in cancer patients who had an intravenous IL-18 injection, while fever was observed only 3 of 21 cases at doses of 100 and 200 μg kg^-1^ [14]. These results indicate the safety of IL-18 for phase I clinical studies in cancer patients [14,15], and a subsequently possible role for cancer-therapy via the immunotherapy concept. Like IL-1, the IL-18 provides its biological function by binding to a specific receptor on the surface of target cell. IL-18 receptor binding sites are divided into the IL-18 receptor α chain (IL-18Rα) (also known as IL-1Rrp1, IL-18R1 or IL-1R5) and the IL-18 receptor β chain (IL-18Rβ) (also termed IL-18RacP, IL-18RII or IL-1R7). The binding sites of IL-18 to its receptors were identified: sites I and II are importantly specific to IL-18Rα and site III to IL-18Rβ [16]. Site I includes Arg13, Asp17, Met33, Asp35 and Asp132; Site II consists of Lys4, Leu5, Lys8, Arg58, Met60 and Arg104, and site III involves Lys79, Lys84 and Asp98. Some other residues (Glu6, Lys53 and Thr63) are also important in the bioactivity and binding mode of IL-18 [17,18].

In this study, we engineered IL-18 by the altering binding residues in order to improve its activity using single point mutagenesis. Since Met33 and Met60 in sites I and II are surrounded by other charged amino acids such as Asp, Arg and Lys, a methionine was replaced by a glutamine to increase the site polarity, based on an implication that these sites require dipole/electrostatic forces to facilitate IL-18 binding. Moreover, according to the report that a substitution of E6 with an alanine [17] and a lysine [19] can enhance IL-18 activity, E6K was also taken in this study as a comparative standard. T63 is another interesting residue, reported for increased activity when it was alanine-substituted [18]. We also combined both types of previous activity enhanced mutations for evaluating the synergistic effect acquired from the polarity change (E6K) and increasing flexibility (T63A). All types of recombinant plasmid were transformed into yeast and the secreted heterologous proteins were purified. The activity was investigated via cell culture and interferon-γ induction which the latter was measured as an immuno-indicator for its anti-cancer immunologic property. Apart from protein engineering and immunologic measurement, molecular dynamics simulations of wild-type and mutated IL-18 were carried out along with experiments to see what impact the point mutation had on overall IL-18 structure and function.

## Materials and Methods

### Cloning and mutagenesis

Total RNA was extracted from colon tissue sample and cDNA was prepared with a Maxima H Minus First Strand cDNA Synthesis Kit (Thermo Scientific). Mature sequence of IL-18 was amplified using the primers listed in Table 1. The first round of product that was tagged with partial TEV protease cleavage site at C-terminus for removing 6xHis tagged after expression was amplified using primer IL-18Fw/IL-18Rv1. This product was then used as a template for complete TEV cleavage site amplification in the second round with another primer set, IL-18Fw/IL-18Rv2 (Table 1). After that, the DNA fragment was ligated into a pTZ57R/T cloning vector (Thermo Scientific) and transformed into competent cell of *E*. *coli* DH5α. Transformants were grown in LB broth containing 100 μgmL^-1^ of ampicillin and plasmid pTZIL18 was extracted and the IL18 mature sequence was confirmed by automated DNA sequencer. To construct the yeast expression vector pPICZα-IL18WT, the mature sequence of IL-18 was sub-cloned into pPICZαA (Invitrogen) at the *Eco*RI and *Xb*aI sites and then transformed into *E*. *coli* DH5α. The transformants were selected on LB medium containing 25 μgmL^-1^ of Zeocin (Invitrogen). A plasmid was extracted and the nucleotide sequence of the mature IL-18 was confirmed by DNA sequencing.

**Table 1:** Primers used in this study.

Primer name	Primer sequence
IL-18Fw	5’-GAATTCTACTTTGGCAAGCTTGAATCTAAATTATCAG-3’
IL-18Rv1	5’-*GTAGAGGTTCTC*GTCTTCGTTTTGAACAGTG-3’
IL-18Rv2	5’-TCTAGATA*TCCCTGGAAGTAGAGGTTCTC*GTCTTC-3’
Pic-FF	5’-TTGTCAGGAACACGATGAAACCAGGATGCC-3’.
E6K-Fw	5’-TACTTTGGCAAGCTT*AAG*TCTAAATTATCAGTC-3’
E6K-Rv	5’- GACTGATAATTTAGA*CTT*AAGCTTGCCAAAGTA-3’
M33Q-Fw	5’-CCTCTATTTGAAGAT*CAG*ACTGATTCTGACTG-3’
M33Q-Rv	5’- CAGTCAGAATCAGT*CTG*ATCTTCAAATAGAGG-3’
M60Q-Fw	5’- AGCCAGCCTAGAGGT*CAG*GCTGTAACTATCTC-3’
M60Q-Rv	5’- GAGATAGTTACAGC*CTG*ACCTCTAGGCTGGCT-3’
T63A-Fw	5’- AGAGGTATGGCTGTA*GC**A*ATCTCTGTGAAGTGT-3’
T63A-Rv	5’- ACACTTCACAGAGAT*TGC*TACAGCCATACCTCT-3’

The underlined bases are recognition sites for *Eco*RI and *Xb*aI, respectively. The italicized sections of the bases are the codons translated for the Tobacco Etch Virus (TEV) protease cleavage site. Base mutations are italicized and underlined.

**Fig 1:**
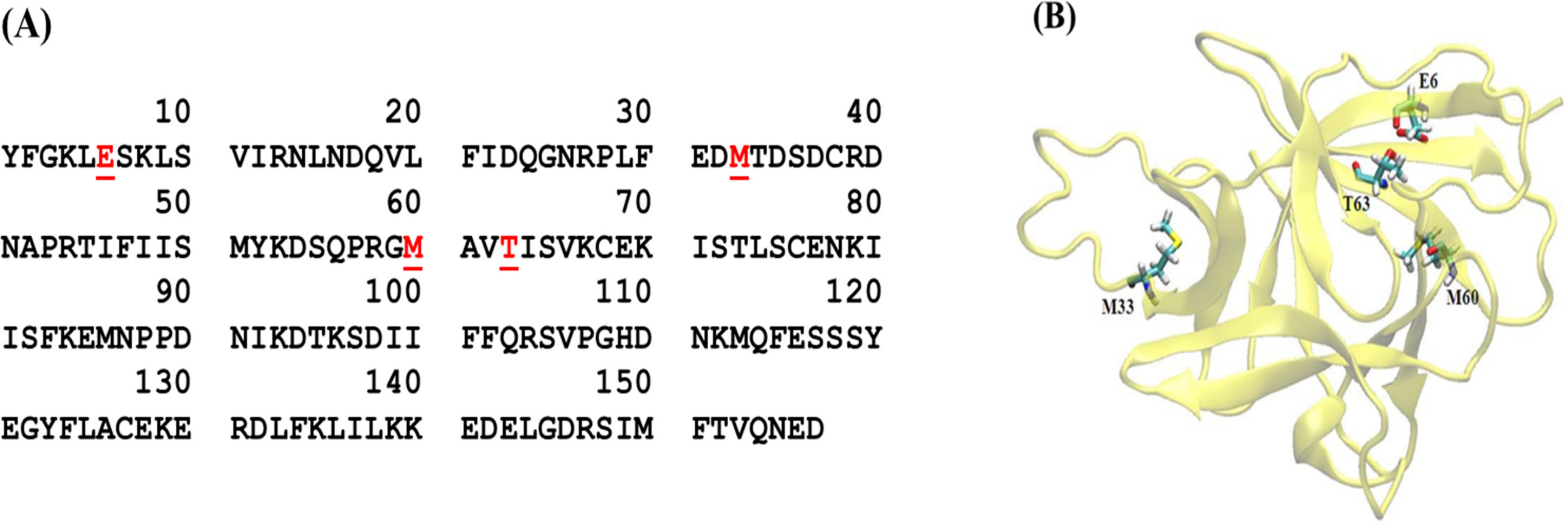
Sequence and structure of human interleukin-18. (A) Amino acid sequence of mature human IL-18 (IL-18). The red and underlined residues are the target amino acid residues that were mutated in this study. (B) 3D structure of mature human IL-18. The residues used in the experiment are shown.

The mutagenesis was performed based on a previous report that divided the binding sites of IL-18 into three regions [16]. To create higher binding affinity to its receptors based on increases in the major force at target sites, the substitutions were performed at the following sites in the IL-18 mature form: E6K, M33Q, M60Q and T63A (Fig 1A). The plasmid pPICZα-IL18WT was used as a template for the mutagenized PCR by using the primer indicated in Table 1. For E6K+T63A, the template plasmid was pPICZα-IL18E6K. The nicked mutagenized plasmids were amplified with Platinum®Pfx DNA polymerase (Invitrogen) and purified. To remove the plasmid template, the purified PCR fragments were digested with *Dpn*I (Thermo Scientific) and transformed into *E*. *coli* DH5α. The transformants were selected and plasmids were verified as described above.

### Protein expression

Plasmid pPICZα-IL18WT and other mutagenized plasmid were linearized with *Sac*I (Thermo Scientific), purified and introduced to *Pichia pastoris* KM71 by electroporation (Invitrogen). The transformants were selected on YPD medium containing 100 μg mL^-1^ of Zeocin (Invitrogen) and plasmid integration was verified by PCR method with the IL-18Rv2 and Pic-FF primers (Table 1). Protein expression was performed by preparing the yeast inoculum in YPD broth and incubated at 30°C at 250 rpm overnight. Then the inoculum was transferred into 200 mL BMGY medium (Invitrogen) with an initial OD_600_ of 0.2 and incubated at 30°C at 250 rpm until the OD_600_ reached 5–6. The cell was concentrated and cultured in 20 mL BMMY medium (Invitrogen) containing 2% methanol at 30°C at 250 rpm for 48 hours. To maintain the induction, methanol was added every 24 hours to give a final concentration of 2%. The supernatant was collected to confirm the existence of recombinant IL-18 by SDS-PAGE and Western blotting.

### Protein purification

The secreted IL18 protein was purified by HisTrap HP column (GE Healthcare) according to manufacturer’s protocol. Briefly, the culture supernatant was loaded into a 1 mL size Ni^2+^Sepharose HisTrap affinity column equilibrated with a binding buffer at pH 7.4 that contained 20 mM sodium phosphate, 0.5 M NaCl and 20 mM imidazole. The native proteins were washed out with washing buffer that contained 50 mM imidazole and the target protein was eluted with buffer containing 400 mM imidazole. The recombinant protein was concentrated by Amicon® Ultra4 centrifugal filter unit (Millipore) and diluted in PBS. Protein concentration was determined spectrophotometrically according to Bradford using bovine serum albumin (BSA) as a standard.

### Western blot analysis

The samples were run on 12% SDS-polyacrylamide gels and transferred onto a polyvinylidene difluoride (PVDF) membrane (Bio-Rad). After electroblotting at 35 volts for 16 hours in transfer buffer using a Transblot unit (Bio-Rad), the target protein was blocked by incubating for 1 hour in TBST containing 3% (w/v) BSA (Sigma-Aldrich), followed by detection with specific mouse anti-IL-18 (R&D systems) in 1:3,000 dilution and incubated at room temperature for 1 hour. The antibody was removed and the PVDF membrane was washed three times for 5 min each in TBST with gentle agitation. Horse radish peroxidase-conjugated goat anti-mouse (R&D system) was added at a dilution of 1:10,000 in TBST containing 3% (w/v) BSA and incubated for 1 hour with gentle agitation at room temperature. The sheet was then washed three times in TBST and antigen-antibody complexes were detected by the addition of LuminataTM Forte Western HRP substrate (Millipore).

### Molecular Dynamic simulation

A structure of human mature IL-18 (157 amino acids) was obtained from the RCSB protein data bank (www.rcsb.org), PDB identification code 1J0S (Fig 1B) [16]. As 1J0S.pdb is an NMR structure, the 3^rd^ conformer was chosen on the basis of it being the lowest RMSD among 20 conformers. All hydrogen atoms in the structure were then removed and the protonation state of amino acid at pH 7 was determined using PROPKA webtools [20]. The missing hydrogen atoms, with corrected protonation state, were then re-inserted using the Leap module in AMBER12 package [21,22]. Six IL-18 mutants (E6K, M33Q, M60Q, T63A, E6K+T63A and M33Q+M60Q) were prepared using the Visual Molecular Dynamics (VMD) package [23] and the Leap module as auxiliary tools. All IL-18 protein was finally energy-minimized using the steepest descent method, under AMBER10 nonpolarizable force field parameters, for 2000 steps.

The minimized protein was neutralized by either sodium (Na^+^) or chloride (Cl^−^) ion and solvated by TIP3P water molecules along with NaCl, yielding a concentration of 0.15 mol dm^-3^. This protein-solution system was equilibrated in an isothermal ensemble (NVT), using Langevin Dynamics as a thermostat set at 310 K (37°C). The harmonic potential was applied to all atomic positions of IL-18 with force constants of 200, 100, 50, and 20 kcal mol^−1^Å^2^, and a time step of 1 femtosecond (fs). The system was finally switched to an isobaric/isothermal (NPT) ensemble with a time step of 2 fs. A temperature of 310 K and pressure of 1.013 bar (1 atm) were regulated using a weak coupling algorithm [24], in order to mimic an *in vivo* environment. The NPT simulation was carried out for 70 nanoseconds (ns) as a product run by a Particle mesh Ewald molecular dynamics simulator (PMEMD) to handle electrostatics calculations with a 12-Å cutoff, implemented in an AMBER12 package. The first 50 ns period was omitted as an equilibration phase, and 2000 equidistant snapshots from the last 20 ns simulation were taken for a configurational average and analysis. Simulations of wild-type and 6 mutated ILs followed the identical protocol. All IL-18 resulting structures were analyzed, compared, and visualized using ptraj module and VMD package.

### Interferon-γ inducing assay

The NK-92MI cells were maintained in complete α-MEM medium supplemented with 12.5% FBS and 12.5% horse serum at 37°C in 5% CO_2_ humidified air. For the assays, NK-92MI cells were suspended at 0.5 × 10^6^ cells per ml in complete α-MEM medium and stimulated in 0.2-ml volumes in 96-well plates with 0.5 ng/ml of IL-12 and different concentrations (200, 100, 50, 25 and 12.5 ng mL^-1^) of recombinant IL-18, or the five mutants. After 16–20 h at 37°C in humidified air with 5% CO_2_, the culture supernatants were collected for IFN-γ measurement by the ELISA method (R&D system).

#### Statistical analysis

The data are presented as means ± SD. Student’s t-test was used to evaluate the significance between groups. P values less than 0.05 (*) and 0.01 (**) were considered statistically significant.

## Results

### 1. Vector construction and mutagenesis

**Fig 2:**
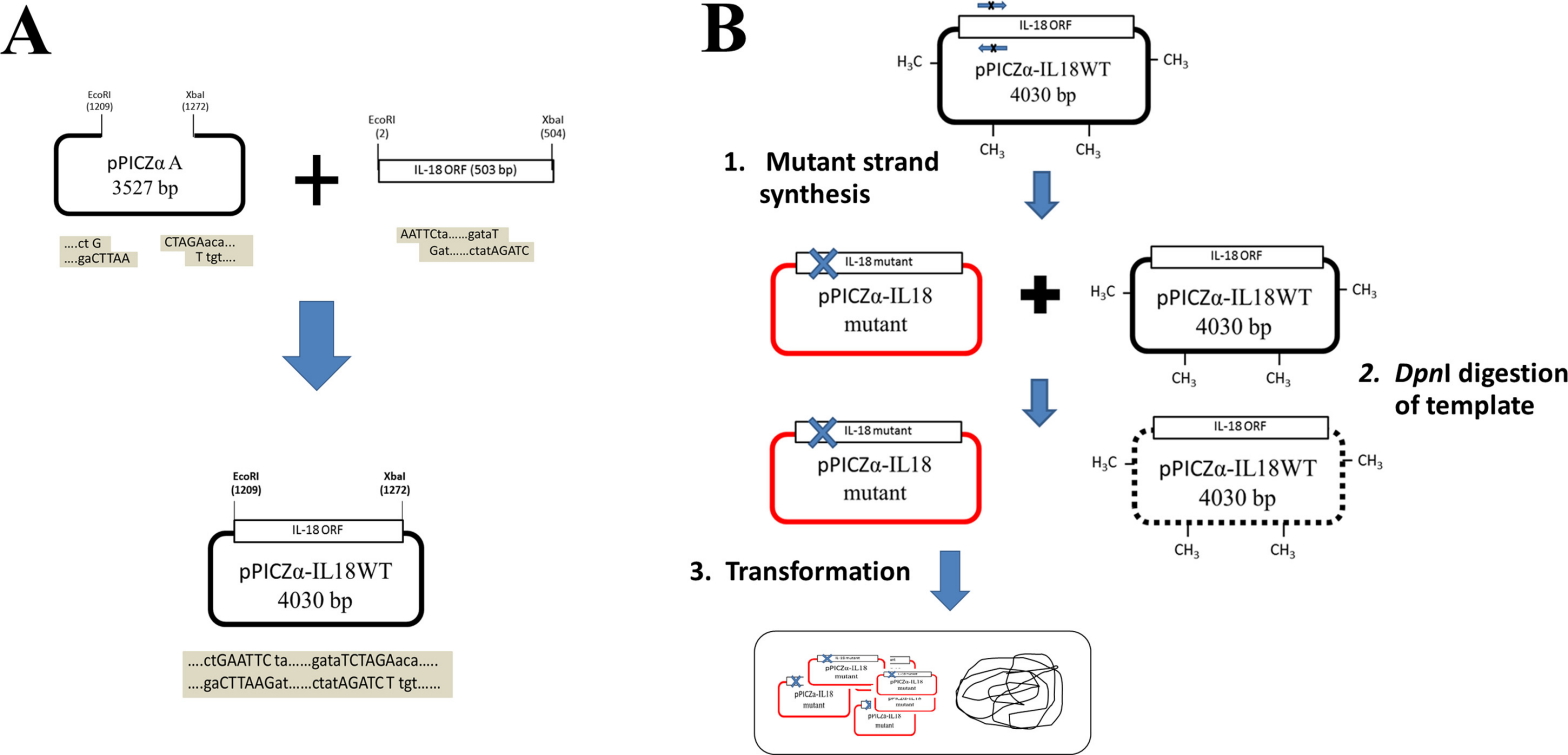
Construction of the expression plasmid pPICZα-IL18WT and its mutants. (A) Strategy and schematic presentation of steps involved in the construction of expression plasmid pPICZa-IL18WT. A mature human IL-18 sequence was inserted into the expression plasmid at the *Eco*RI and *Xba*I sites. (B) Diagram showing the site-directed mutagenesis method. The mutant-strand was amplified by PCR and a wild-type DNA template was digested by *Dpn*I. The resulting annealed double-stranded nicked DNA molecules were transformed into *E*. *coli* DH5α and the nicked DNA was repaired.

The mature sequence of human IL-18 (≈500 bp) was successfully amplified from cDNA of human and cloned into yeast expression vector pPICZαA (Invitrogen). This gene was under the control of methanol inducible *AOX1* promoter and extracellular secretion of protein was mediated by α-mating factor secretion signal derived from *Saccharomyces cerevisiae* (Fig 2A). For site-directed mutagenesis, we chose to modify amino acid binding residues (E6K, T63A) and increase the polarity of the amino acids (M33Q, M60Q) [16]. To construct the mutant plasmids, mutagenesis was achieved by whole plasmid mutagenesis introduced mutation into the wild-type expression vector, pPICZα-IL18WT (Fig 2B). Five mutant plasmids namely pPICZα-IL18E6K, pPICZα-IL18M33Q, pPICZα-IL18M60Q, pPICZα-IL18T63A, and pPICZα-IL18E6K+T63A were accomplished and used to transform yeast *P*. *pastoris* KM71 by electroporation.

### 2. Expression, purification and validation of recombinant IL-18

**Fig 3:**
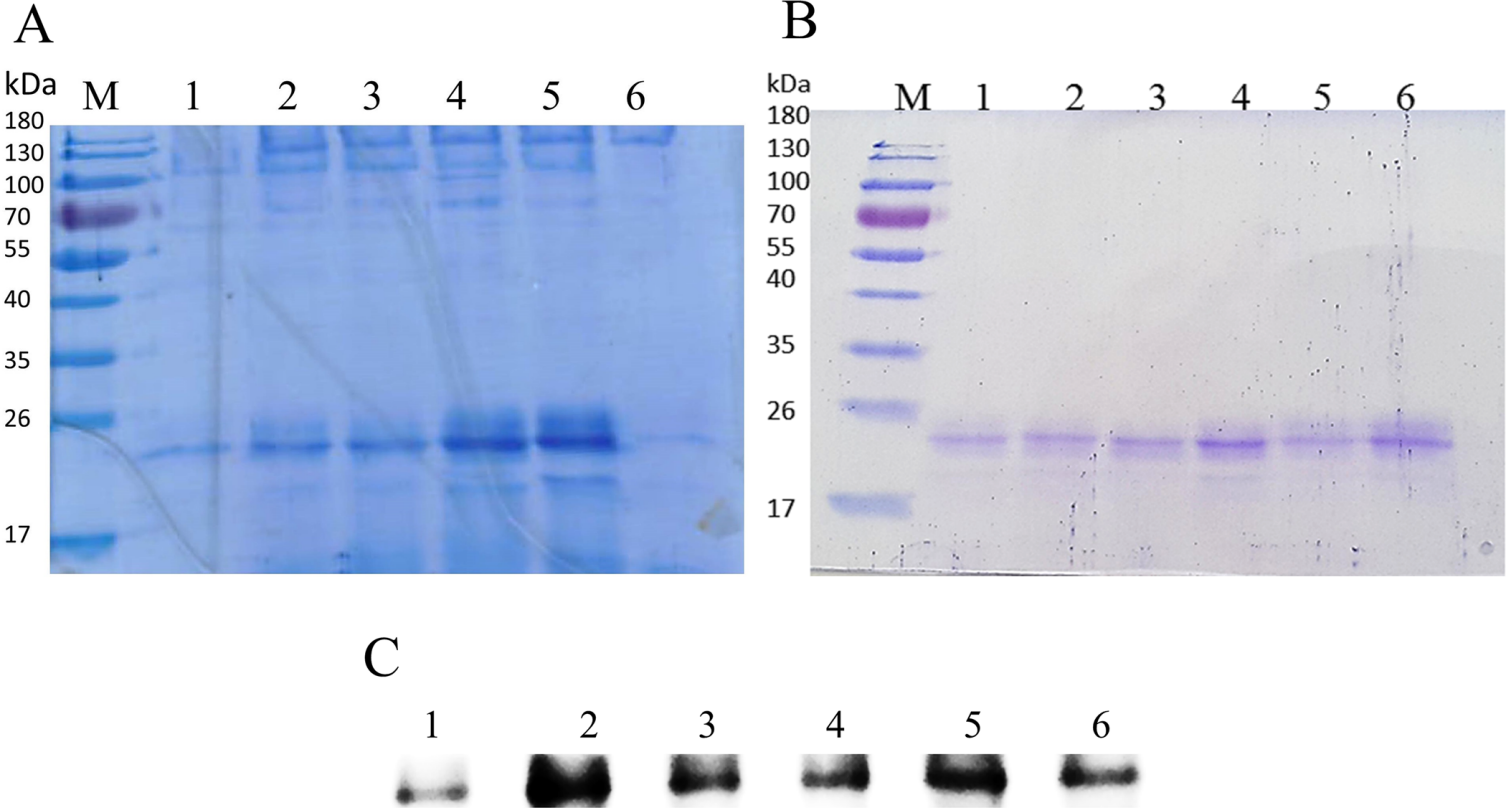
SDS-PAGE and Western blot analysis of recombinant IL-18. (A) SDS-PAGE analysis of secreted proteins from the *P*. *pastoris* KM71 before purification. (B) SDS-PAGE analysis of recombinant IL-18 after purification with HisTrap affinity column, (C) Western blot analysis of recombinant IL-18 protein. All samples were loaded onto 12% SDS-PAGE. Lane: M = Molecular mass standard (Thermo Scienctific), 1 = wild-type, 2 = E6K, 3 = M33Q, 4 = M60Q, 5 = T63A and 6 = E6K+T63A.

**Fig 4:**
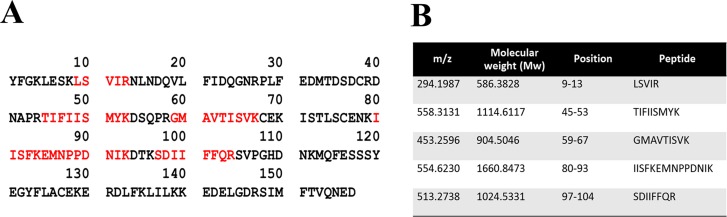
LC-MS/MS analysis of purified recombinant mature IL-18. (A) Tryptic peptide map of recombinant mature IL-18 produced by yeast *P*. *pastoris*. The identified peptides are shown in red, (B) Identified tryptic peptide fragment derived from purified IL-18.

Extracellular expression of recombinant IL-18 was performed in yeast *P*. *pastoris* KM71. The crude proteins secreted by *P*. *pastoris* were analysed by SDS-PAGE. As shown in Fig 3A, a protein band (≈ 22 kDa) that corresponded to mature IL-18 was detected in the transformant harboring an IL-8 expression plasmid. However, this band was not detected in the transformant having an empty plasmid (data not shown). This result suggested that recombinant IL-18 was expressed in yeast *P*. *pastoris*. For further validation of the recombinant protein, Western blot and LC-MS/MS analysis of the purified protein were performed. After purification, a purified protein band (≈ 22kDa) was observed (Fig 3B) and this band was validated as IL-18 by Western blotting (Fig 3C). In addition, the protein was excised from the gel and submitted to Proteomics International Pty Ltd. for LC-MS/MS analysis as well. Tryptic digestion of 22 kDa protein produced 5 peptide fragments that covered 28.66% of the mature IL-18 (Fig 4). These data indicated that the purified protein was mature IL-18.

### 3. Interferon-γ induction assay

**Fig 5:**
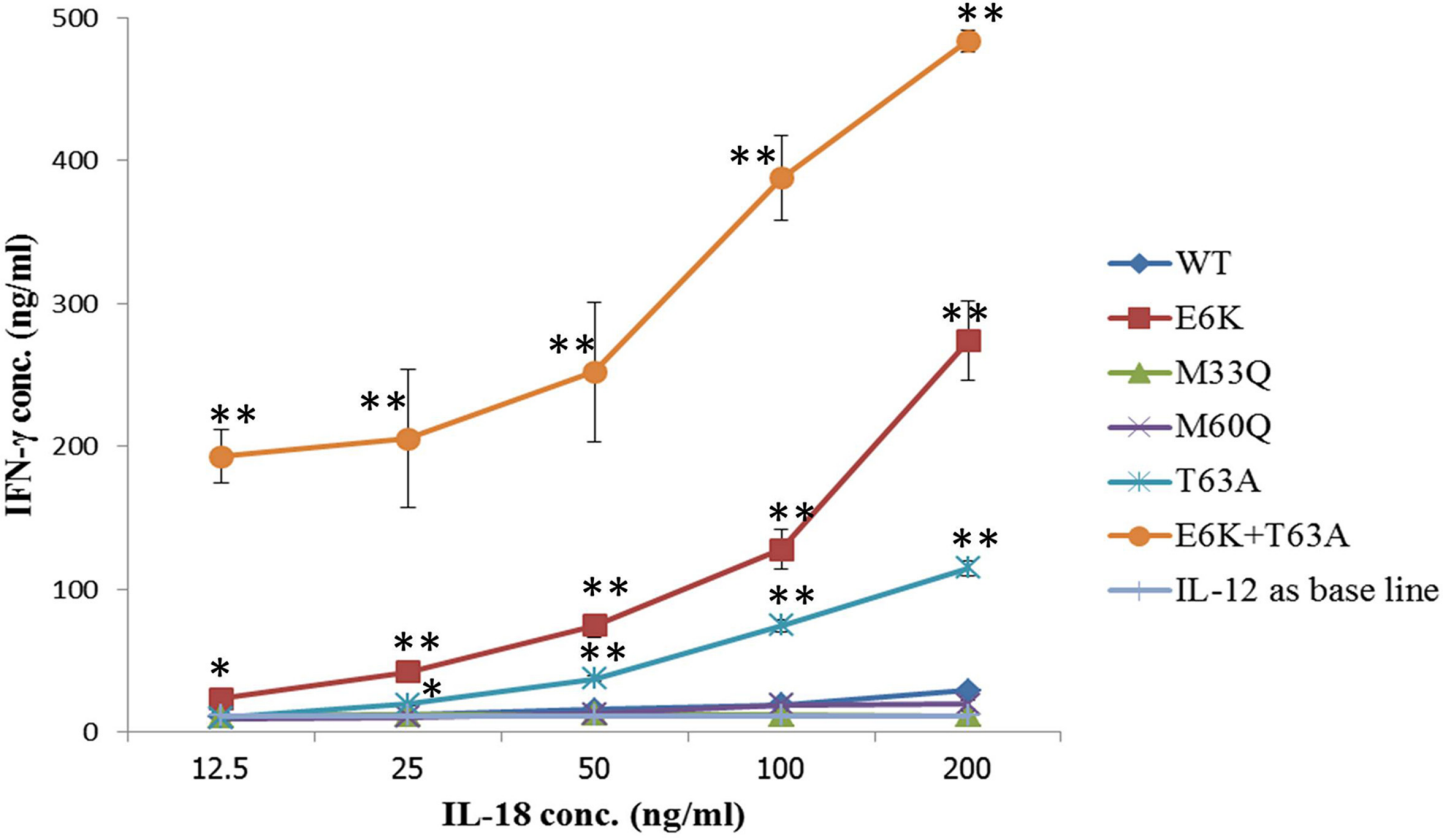
IFN-γ induction assay with the purified recombinant IL-18. NK-92MI cells were treated with various concentrations of recombinant purified IL-18 for 24 hours. IFN-γ was measured in the supernatant by ELISA. The illustrated results represent the mean SD of three independent experiments. *p < 0.05 and **p < 0.01, compared with the levels of IFN-γ that were simulated with wild-type IL-18.

To test the biological activity of wild-type IL-18 and its five mutants, NK92-MI cells were used to measure the ability of the protein to induce IFN-γ in the presence of IL-12 as a co-stimulant. The results showed that at a low concentration (12.5 ng mL^-1^), most IL-18, including wild-type, M33Q, M60Q and T63A, had no influence on IFN-γ production, whereas the E6K and E6K-T63A double mutations could induce significantly different levels of IFN-γ, especially the double mutation had about a 17x greater impact on IL-18 activity than the wild-type. Although WT IL-18 activity was observed at 100 and 200 ng mL^-1^, and M33Q was observed only at 200 ng mL^-1^, the M60Q forms did not show any IFN-γ induction activity at all concentrations (Fig 5). Interestingly, NK92-MI cells could be stimulated to produce IFN-γ by just only 25 ng mL^-1^ of E6K, T63A and E6K+T63A. Obviously, higher levels of activity of E6K, T63A and E6K+T63A were observed when the concentrations of recombinant proteins were increased to 50, 100 and 200 ng mL^-1^. The activities of E6K, T63A and E6K+T63A forms were about 9.3, 3.9 and 16.4 times higher than wild-type IL-18 at a concentration of 200 ng mL^-1^.

### 4. Molecular dynamic simulation

**Fig 6:**
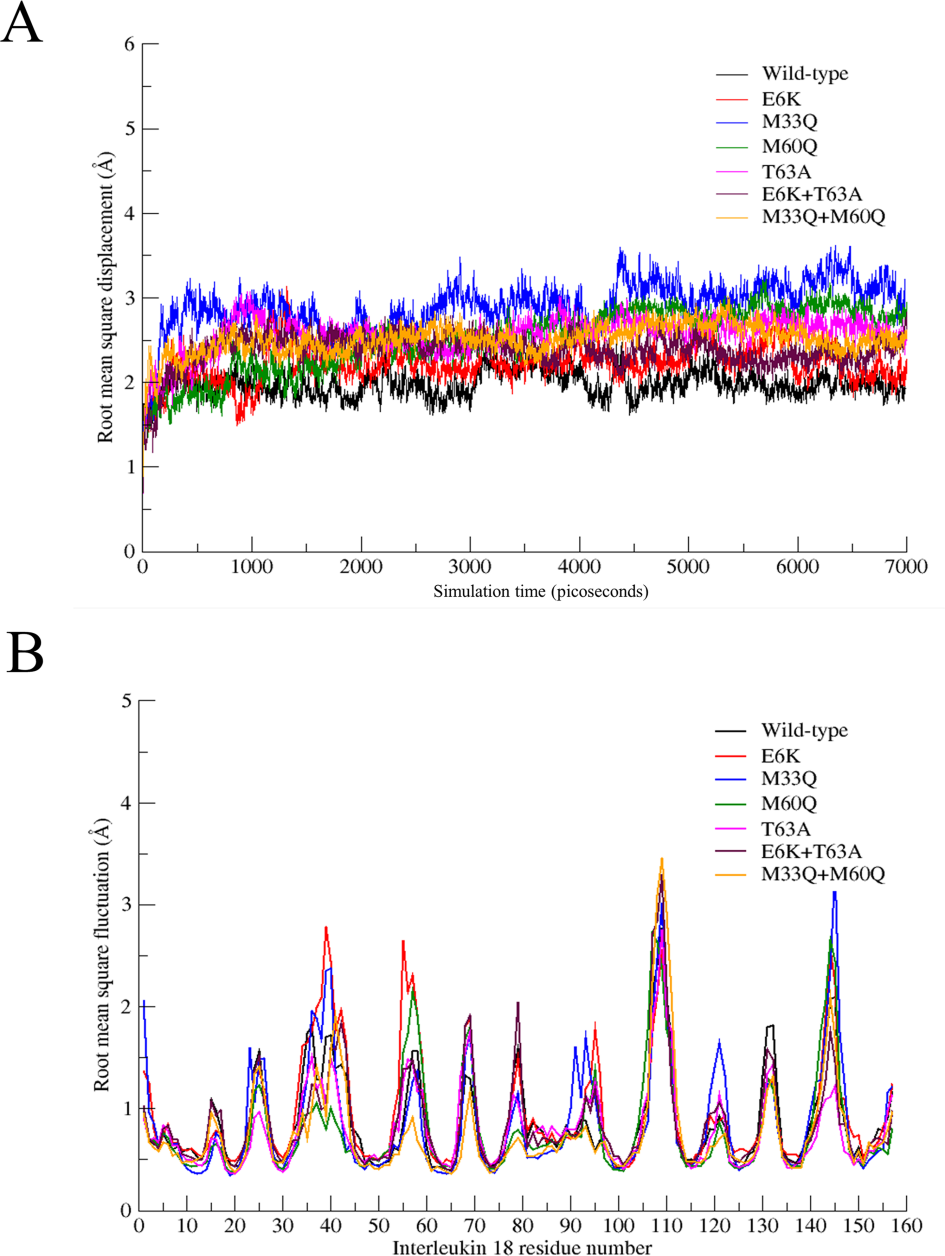
(A) Root-mean-square displacement (RMSD) in Angstrom units from MD simulations of all IL-18 proteins, relative to the initial coordinates, the 3^rd^ conformer of 1J0S NMR structure. (B) Root-mean-square fluctuation (RMSF) from IL-18 MD simulations.

**Fig 7:**
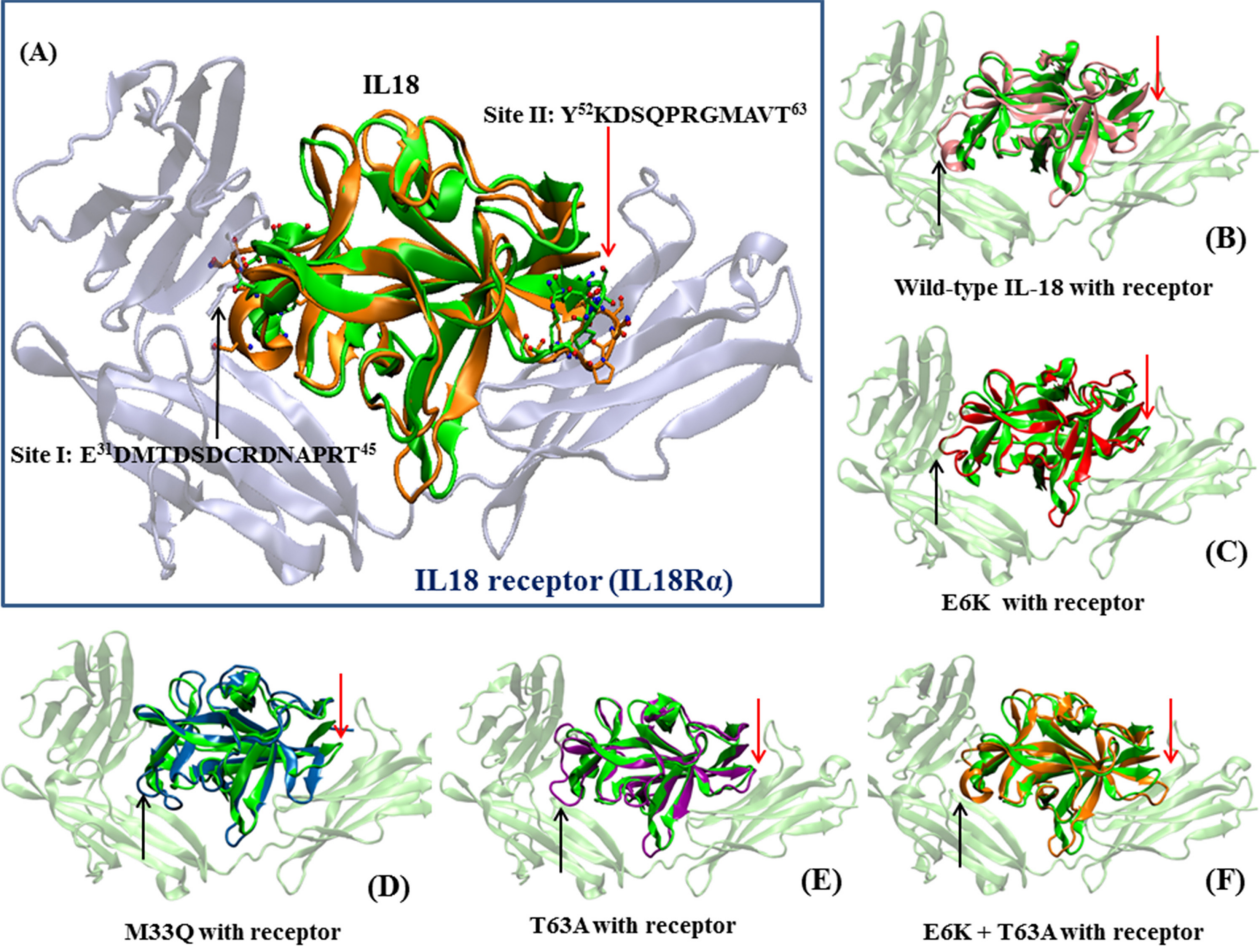
IL18-IL18Rα complex: Fig 7(A) shows the IL18 crystal structure (green) of the complex, aligned with the MD structure of a double-mutated IL18. The residues of IL18 interactions with site I and site II in IL18Rα are indicated. (B) to (G) illustrate all average structures of simulated IL18 with respect to IL18 in the IL18-IL18Rα complex crystal. The red arrows indicate the loop from E31 to T45, which directly interacts with IL18Rα.

The effect of a point mutation on the IL-18 conformation/structure was investigated through a molecular dynamic study of aqueous IL-18 at 37°C, using an NMR IL-18 structure as a molecular template. All IL-18 proteins in a NaCl solution were simulated and later analyzed for structural change with respect to the template. All energetic parameters, such as temperature, pressure, energy and density, became stable throughout the simulations. An MD simulation showed that, in the case of wild-type IL-18, the average dynamic structure differed slightly from the experimental NMR structure, with a root-mean-square-distance (RMSD) of approximately 2 Å, the lowest among all MD simulations (Fig 6A). Significant differences in structure were observed from M33Q, and T63A, (Fig 7), with an RMSD of circa 2.8 Å, whereas E6K and the double mutation lead to similar tertiary IL-18 structures, almost close to a wild type case. In other word, this suggests that the M33Q and T63A mutations caused a vivid conformational alteration in the IL-18 structure, while E6K preserved the tertiary IL-18 structure.

To verify this hypothesis in more detail, an MD simulation of a double-point mutated IL-18 was also carried out. The two mutation sets, E6K+T63A and M33Q+M60Q, were selected to investigate the effect on overall conformation if both mutations were simultaneously present. For E6K+T63A, an RMSD as well as an average structure of E6K+T63A simulation indicated closer similarity to a wild type IL-18, as shown in Figs 6A and 7. This result confirmed that E6K alteration had an impact on conformational conservation in IL-18, even in such a case T63A is located in a structure. Another mutation is M33Q+M60Q is also discussed herein. The M33Q+M60Q overall structure was more similar to the wild type IL-18, compared to the single-point mutated structures (M33Q or M60Q). The simulation suggested that the existence of both M33Q and M60Q might result in a better affinity than M33Q- and M60Q-IL18.

Apart from the structure similarity, conformational flexibility was also observed in order to visualize the dynamic behavior of IL-18. The flexibility was quantified by root-mean-square-fluctuation (RMSF) plotted against protein residue order (1^st^-159^th^ for IL-18), (Fig 6B), with reference to the starting template (1J0S.pdb). All IL-18 proteins share a similar RMSF pattern in most of the entire structure. Some distinct features were however observed in E6K, M33Q and T63A, which we discuss in detail later. Surprisingly, even though E6K did not contribute to a conformational charge, unlike M33Q and T63A, three of them were responsible for more structural flexibility, compared to wild-type IL-18.

## Discussion

Some research studies have revealed that IL-18 has potential as an anti-cancer agent [25,26], however, this knowledge has not yet been successfully translated into the clinical practice. This may be due to the low biological activity of wild type IL-18 caused by the IL-18-binding protein (IL-18BP) which is a constitutively secreted IL-18 neutralizing protein present in healthy individual serum in 20-fold molar excess compared to IL-18 and binds to IL-18 with a high affinity (400pM) *in vitro* and *in vivo* [27–29]. There are many studies underway attempting to find ways to enhance the activity of the protein. It has been reported that increasing both bioactivity and bioavailability of IL-18 was possible by mutagenesis of amino acid residues [17,19,30] and perform protein fusion of IL-18 and IL-2 [30]. Although it is known that biological function of IL-18 involves the binding of protein to a specific receptor and mutation of amino acid binding site can enhance biological activity, there is little information on how mutation has an impact on overall IL-18 structure and function. Thus, this prompted us to investigate the impact of mutation on IL-18 structure.

In this study, the biological activity of IL-18 was enhanced by mutation of amino acids based on their receptor binding residues and polarity and the impact of mutation on the IL-18 structure was elucidated. It was previously reported that M33 in site I, M60 in site II and some other residues (E6, T63) were associated with the binding mode and bioactivity of IL-18 [17,18]. Based on this information, we expected that alteration of some IL-18 binding residues could enhance IL-18 activity. To increase the polarity in site I and site II, M33Q and M60Q were performed. We expected these substitutions would increase the ligand-receptor affinity. Moreover, T63A was achieved in order to increase the flexibility of the recognition loop of the protein and E6K was used as a standard comparison.

Because *P*. *pastoris* secretes only small amounts of endogenous proteins, secretion of recombinant protein constitutes the major protein in the medium that resulted in much easier first step purification [31]. Thus, the *P*. *pastoris* expression system was used for expression of recombinant IL-18 in this study. The recombinant IL-18 proteins were found to secrete by yeast *P*. *pastoris* with a high yield (approximately 1–4 mg mL^-1^), which was purified to homogeneity by a HisTrap affinity column and used for the assays of biological activity.

In our study, at the concentration of 200 ng mL^-1^, E6K, T63A and E6K+T63A exhibited higher IFN-γ inducing activity with factors of 9.3, 3.9 and 16.4 respectively, compared to wild-type IL-18. For the mutants M33Q and M60Q the activity was however slightly lower. These data clearly indicate that all performed mutations affect the affinity of IL-18 for its receptor, via changes in either structure or interaction. Recently, after our experiments had been carried out, a recognition model between IL-18 and its receptor was crystallized and reported [32] (pdb code 3WO3). We applied this model structure along with our MD simulations to describe how E6K, T63A and E6K+T63A mutations influenced the ligand-receptor binding affinity.

**Fig 8:**
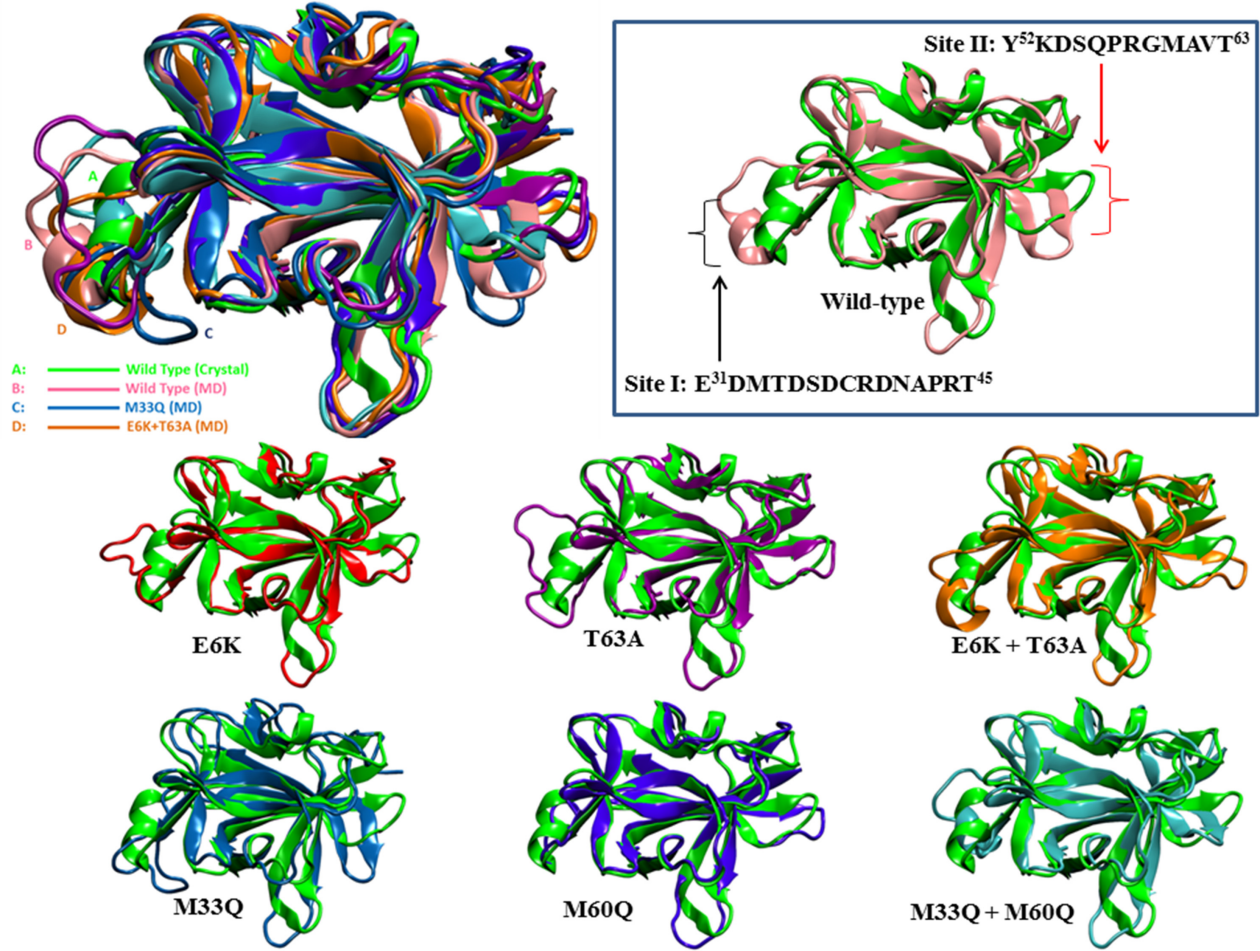
Comparison between an average structure from MD simulation and the reference experimental NMR structure (PDB code 1J0S)(Green): The arrows indicate the loops from E31 to T45, which directly interact with IL-18Rα site I, and from Y52 to T63, the interface of IL18 and site II in the receptor structure, respectively.

Structurally, when all mutation trials were performed, there were 2 important regions that were changed. The first one was the loop that contains the E^31^DMTDSDCRDNAPRT^45^ sequence which some of these residues seemed to play a role in site I interaction at the IL-18Rα pocket [32] (Fig 7). Structural alignment (Fig 7) revealed slightly different kinds of conformational alteration in each type of mutation that may have been responsible for the activity changes. For example, E6K and E6K+T63A showed small conformational changes at this area which may have facilitated IL-18 binding to the receptor, especially D37, R39, D40 and N41 that changed directly forward to the IL-18Rα pocket (Figs 7 and 8). Specifically, since root mean square fluctuation (RMSF), responsible for protein flexibility, of E6K at this loop is obviously higher than other types of mutations (Fig 6B), this can indicate that the increasing flexibility may give a higher possibility of this loop to adapt itself to the appropriate structure for receptor binding.

**Fig 9:**
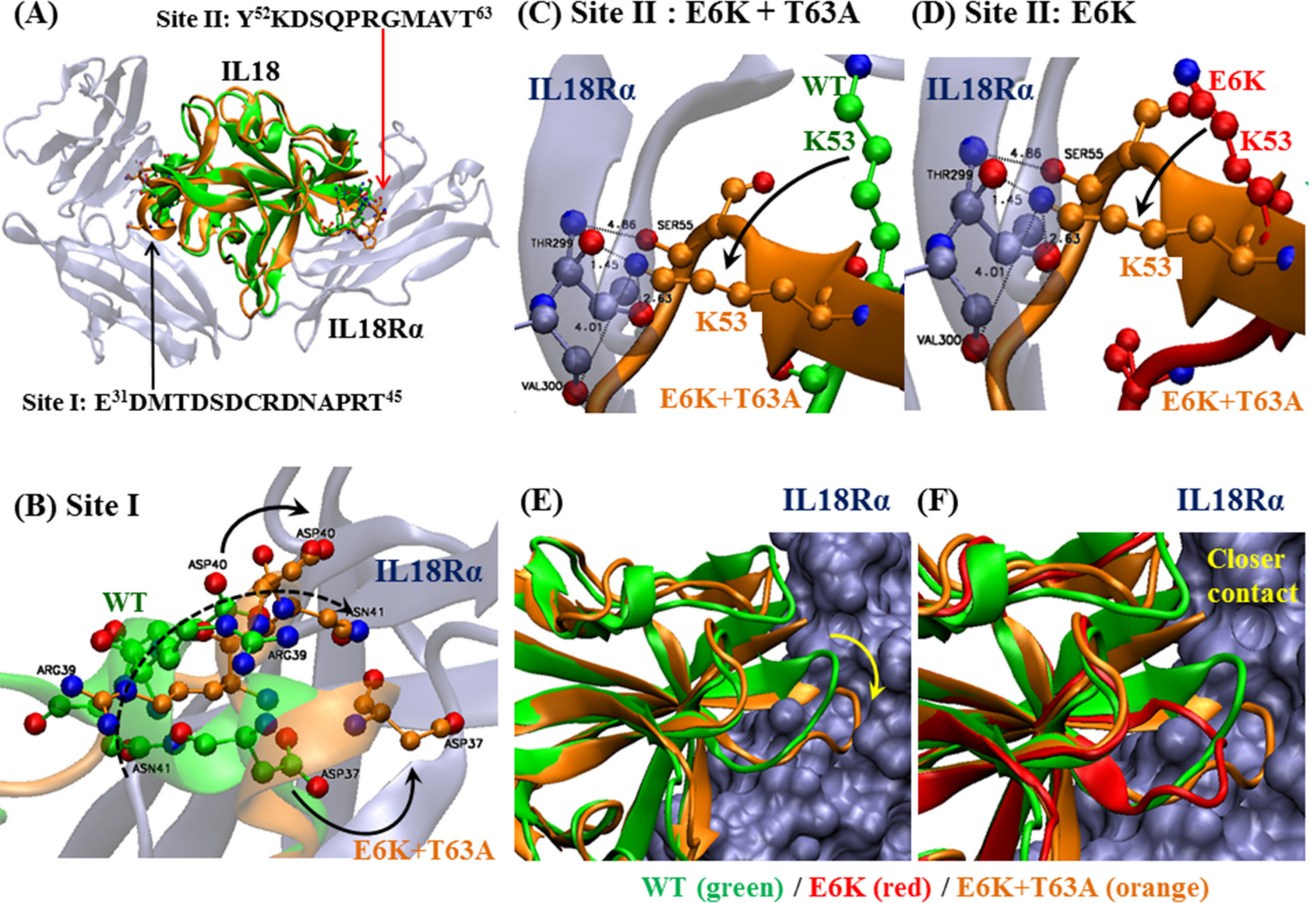
Interaction of mutant IL18 with IL18Rα at site II: Fig 8(A) shows the site II interface between IL18 and IL18Rα. Fig 8(B) shows a comparison of the IL18 structure at site II between WT IL18 (green) and E6K+T63A IL18 (orange). The mutated IL18 structure (orange) clearly comes closer to site II. Fig 8(C) shows the molecular interaction between the protein and receptor. In the mutant, K53(IL18) moves to bind to T299 and V300 of the receptor, whereas in the wild-type structure, this K53 resides far from these two residues. Finally, Fig 8(D) shows E6K+T63A mutation facilitate the loop in the IL18 to make closer contact with the receptor, compared to the wild-type and E6K cases.

M33Q, M60Q and T63A showed larger structural alterations with little difference in RMSDs. However, the activity of T63A increased while decreasing in the others. This may be due to different types of loop changes since M33Q showed malformation of the loop structure. In M33Q, the direction of loop was changed whereas T63A demonstrated a new loop direction towards the receptor (Figs 8, 9(B) and 9(D)).

The second important region is the Y^52^KDSQPRGMAVT^63^ loop since this loop consists of functional K53, a key residue for receptor binding site II of IL-18 [32]. The activity of IL-18 may be affected some alterations occur at this region. The MD simulation revealed that this loop was most affected by M33Q as the secondary structure of this loop was transformed into a sheet resulting in the abolishing of site II interaction (Fig 9). The activity loss of M33Q may not only be from this interaction elimination, but also from the intra-structural changes. As methionine tends to reside in buried hydrophobic regions, it tends to serve as a stabilizing protein core structure, rather than a reactive functional residue [33,34]. This is in good agreement with the molecular dynamics results in which M33Q could give structural destabilization and disturb hydrophobic buried structures. Apart from T63, another mutation affecting this loop is E6K, although molecular dynamics simulation revealed wild-type IL-18 and E6K shared a similar general structure, namely β-sheets and α-helices (Figs 6A and 9). However, although the flexibility of this loop was increased and the loop structure showed a new conformation when compared to wild-type (Figs 6B and 8), the activity was not disrupted. This may be because the higher flexibility of this loop could cause the increasing possibility of K53 to contact with the corresponding amino acid in IL-18Rα. Moreover, the E6-surrounding region of IL-18Rα contains acidic residues (E244, E245 and D246). The distance between the residues to E6 is approximately 5.4–7.5 Å. It is possible that lysine substitution at E6 in IL-18 can promote binding affinity to the receptor via increasing both cationicity and surface area. With this overall perspective, we can surmise that the increasing activity of the E6K mutation was mostly regulated by charge transformation at the E6 residue that caused the small conformational change at the first and second regions described above leading to the better binding of IL-18 to its receptor.

Interestingly, when E6K and T63A were combined, the activity of this combination was highest among all other types of mutations. Moreover, the RMSD value was reduced to near the level of the wild-type protein, suggesting that there was no significant difference in E6K+T63A structures, with respect to wild-type IL-18. However, the synergistic activity expressed from a double-point mutated IL-18, with E6K and T63A can be derived from a small change of the protein at the loop that contains K53 as a key residue (Figs 8, 9(A) and 9(B)). Molecularly, the loop change caused a novel direction of K53 which could lead to the interaction of K53 of IL-18 to T299 and V300 deeply in the IL-18Rα molecule (Fig 9(A) and 9(C)). D54, S55, Q56, R58 and G59 were also affected by the loop change which may be due to the combination of the mutations, shown in Fig 9(B). This resulted in the reorganization of mentioned amino acids and led to the forming of a special interaction of these molecules to glycan of N297 of IL-18Rα which cannot be found in E6K, Fig 9(D). Since *N*-linked glycosyl chain in N297 of IL-18Rα close to the β4-β5 loop of IL-18 is responsible for the important bridging of the D3:D3 domains of IL-18 and IL-18Rα through electrostatic and hydrophobic interactions [32], this scenario makes this type of mutation show a synergism between E6K and T63A.

## Conclusion

In summary, we successfully produced IL-18 protein using *P*. *pastoris*. Moreover, we showed that the production is so simple and efficient that it may facilitate further studies of IL-18 in the near future. In addition we have demonstrated that point mutations of IL-18 truly affected its biological function and immunologic functions should be able to be regulated through this approach. E6 and T63 can be modified for improving the activity of this protein to some extent and synergistic effects are observed when these two mutations are combined. Meanwhile, IL-18 activity slightly dropped when some positions such as M33 and M60 were mutated, implying these crucial residues need to be preserved for efficient IL-18 function. Our study has also given molecular insights into how mutations influence IL-18 activity both in terms of 1) effect on protein conformation and flexibility, and 2) intra-protein and protein-receptor interactions. Further studies regarding synergism of IL-18 activity due to simultaneous multiple mutations as well as extended studies for possible immunological activity from IL-18 with additional point mutations can enlarge our understanding of how IL-18 modulates immunologic function via receptor binding.
